# The role of social support in antiretroviral therapy uptake and retention among pregnant and postpartum women living with HIV in the Greater Accra region of Ghana

**DOI:** 10.1186/s12889-024-18004-z

**Published:** 2024-02-21

**Authors:** Edward Kwabena Ameyaw, Jerry John Nutor, Jaffer Okiring, Isaac Yeboah, Pascal Agbadi, Monica Getahun, Wisdom Agbadi, Rachel G.A. Thompson

**Affiliations:** 1 Institute of Policy Studies and School of Graduate Studies, Lingnan University, Hong Kong, China; 2 Africa Interdisciplinary Research Institute, Accra, Ghana; 3 L & E Research Consult Ltd, Wa, Upper West Region, Ghana; 4 Department of Family Health Care Nursing, School of Nursing, University of California San Francisco, San Francisco, CA USA; 5 Infectious Diseases Research Collaboration, Kampala, Uganda; 6 Institute of Work Employment and Society, University of Professional Studies, Accra, Ghana; 7 Department of Sociology and Social Science Policy, Lingnan University, Hong Kong, China; 8 Institute for Global Health Sciences, University of California, San Francisco, San Francisco, CA USA; 9 Push Aid Africa, Accra, Ghana; 10 Language Center, College of Humanities, University of Ghana, Accra, Ghana

**Keywords:** HIV, Antiretroviral therapy adherence, Social support, Pregnant women, Postnatal, Ghana

## Abstract

**Introduction:**

The role of social support in antiretroviral therapy (ART) uptake and retention among pregnant and postpartum women in Ghana’s capital, Accra, has received limited attention in the literature. This cross-sectional study extends existing knowledge by investigating the role of social support in ART adherence and retention among pregnant and postpartum women in Accra.

**Methods:**

We implemented a cross-sectional study in eleven (11) public health facilities. Convenience sampling approach was used to recruit 180 participants, out of which 176 with completed data were included in the study. ART adherence in the three months preceding the survey (termed consistent uptake), and ART retention were the outcomes of interest. Initial analysis included descriptive statistics characterized by frequencies and percentages to describe the study population. In model building, we included all variables that had *p*-values of 0.2 or lesser in the bivariate analysis to minimize negative confounding. Overall, a two-sided *p*-value of < 0.05 was considered statistically significant. Data were analyzed using Stata version 14.1 (College Station, TX).

**Results:**

In the multivariate model, we realized a lower odds trend between social support score and consistent ART adherence, however, this was insignificant. Similarly, both the univariate and multivariate models showed that social support has no relationship with ART retention. Meanwhile, urban residents had a higher prevalence of ART adherence (adjusted Prevalence ratio (aPR) = 2.04, CI = 1.12–3.73) relative to rural/peri-urban residents. As compared to those below age 30, women aged 30–34 (aPR = 0.58, CI = 0.34–0.98) and above 35 (aPR = 0.48, CI = 0.31–0.72) had lower prevalence of ART adherence Women who knew their partner’s HIV status had lower prevalence of ART adherence compared to those who did not know (aPR = 0.62, CI = 0.43–0.91). Also, having a rival or co-wife was significantly associated with ART retention such that higher prevalence of ART adherence among women with rivals relative to those without rivals (aOR = 1.98, CI = 1.16–3.36).

**Conclusion:**

Our study showed that social support does not play any essential role in ART adherence among the surveyed pregnant and postpartum women. Meanwhile, factors such as having a rival and being under the age of thirty play an instrumental role. The study has signaled the need for ART retention scale-up interventions to have a multi-pronged approach in order to identify the multitude of underlying factors, beyond social support, that enhance/impede efforts to achieve higher uptake and retention rates.

**Supplementary Information:**

The online version contains supplementary material available at 10.1186/s12889-024-18004-z.

## Introduction

Despite the global commitment to combating Human Immunodeficiency Virus (HIV), it remains a major public health threat. Globally, a total of 39 million people were living with Human Immunodeficiency Virus (HIV) in 2022, with about 40.1 million associated deaths since its emergence [1]. There is significant variation across countries and regions and globally, the World Health Organisation (WHO) African Region is the hardest hit, as almost 1 in every 25 adults (3.4%) living with HIV reside in the region [1]. This signifies over two-thirds of the persons living with HIV globally.

The primary mode of transmission of HIV in Ghana is heterosexual intercourse (accounting for 75%-80% of all transmissions), followed by blood transfusion and vertical transmission from mother to child, with both accounting for about 20% [2, 3]. HIV is generally high among key populations which include men who have sex with men (MSM) and female sex workers (FSW) [4]. Ghana is committed to the global pursuit to ending HIV by the year 2030 [5]. Consequently, several interventions have been implemented. Thus, through agencies like the Ghana AIDS Commission, the nation has implemented several interventions including HIV/AIDS testing [6].

Over the years, successive governments have demonstrated strong commitment to combating HIV through diverse approaches and interventions, including antiretroviral therapy (ART) [6]. The 2022 Consolidated Guidelines on HIV care differentiates ART initiation by persons presenting to care when clinically well (Stage 1 and 2 and CD4 > 200 cells/mm3) or persons presenting to healthcare with advanced HIV (Stage 3 or 4 and/or CD4 < 200 cells/mm3) [7]. The WHO also accentuates the need to initiate ART for all people living with HIV regardless of WHO clinical stage and at any CD4 cell count [8].

To guarantee that the patient is aware of the requirements for taking ART and can make an informed decision about starting and adhering to lifelong treatment, the patient must attend at least two sessions of adherence counseling before beginning treatment [2]. Considering the stigma and other negative interpretations ascribed to HIV, patients seeking ART may require some social support from their partners, friends, family members and significant others [9, 10]. For instance, evidence from Uganda suggests that support from family and caregivers enhances ART adherence [11]. Typically, people living with HIV who get others to remind them to take ART or go for refill tend to have higher adherence [12].

The association between social support and antiretroviral therapy (ART) adherence among HIV patients has been explored in empirical studies [12]. Theoretical assumptions suggest a positive relationship, indicating that increased social support should enhance adherence rates. However, existing research has yielded conflicting findings, with some studies supporting this assumption [13, 14], while others have reported negative associations [15, 16] or no significant relationship at all [11]. These inconsistencies necessitate a deeper investigation into the complex interplay between social support and ART adherence, especially in a different social group of HIV patients. Therefore, this study aims to fill this research gap by reexamining the hypothesized relationship between social support and ART adherence among HIV-positive pregnant women (prenatal) and HIV-positive women (post-partum) in Ghana’s capital, Accra. Hence, the objective of the study was to investigate the association between social support and ART adherence among HIV-positive pregnant women (prenatal) and HIV-positive women (post-partum). By focusing on a different social group, we aim to provide a nuanced understanding of the complex relationship between these variables. By examining the relationship between social support and ART adherence in a social group, this study will provide valuable insights into how the association may vary across different populations of persons living with HIV.

Our extensive search revealed that the role of social support in ART adherence and retention among pregnant and postpartum women in Ghana’s capital, Accra, has received limited attention in the literature. This cross-sectional study, therefore, sets out to extend existing knowledge by investigating the association between social support and ART adherence and retention among pregnant and postpartum women in Accra, Ghana. The findings of this study will offer a deeper understanding of whether social support enhances or inhibits the uptake and retention of ART, inform the Ministry of Health and the Ghana AIDS Commission about social support for pregnant and postpartum women living with HIV, thereby safeguarding the wellbeing of birthing mothers and their newborns. Besides, the findings will be useful to researchers whose research interest lies in HIV care and social support.

## Theoretical framework

This study is anchored in the Social Support Theory (SST) [17]. The main tenet is that the possibility of delinquency and crime is decreased by instrumental, informational, and emotional assistance. Providers of social support include family, friends, and the community’s capable members who are always willing to help. These networks are shaped by the people, events, and situations that are going on in the world now, specifically for HIV-positive pregnant women (prenatal) and HIV-positive (post-partum) women. This framework posits that social support is a dynamic construct that is contingent upon the unique needs and circumstances of those in need of it) [17], thus HIV-positive pregnant women (prenatal) and HIV-positive (post-partum) women in the context of this study. According to the framework, an individual’s capacity to obtain social support is contingent upon personal attributes, which encompass their age, gender, and living situation in addition to their relationship status and other situational factors like their own expectations, resources, and demands. Underpinned by this framework, we investigated the association between social support and ART adherence among HIV-positive pregnant women (prenatal) and HIV-positive women (post-partum).

## Methods

### Study setting

We implemented this study in the Greater Accra region of Ghana. The region is the smallest of the 16 administrative regions in Ghana, making just 1.4% of the country’s total land area [18]. The 2021 Population and Housing Census indicated that the region has a total population of 5,455,692 consisting of 2,679,063 males (49.1%) and 2,776,629 females (50.9%) [19]. Between 1984 (441) and 2000, the population density of the area doubled, partly due to migrant flows into the area. The region has a network of health institutions, including 707 CHPS facilities, 299 clinics, 101 maternity homes, 32 health centers, 22 polyclinics, and 111 hospitals, which provides healthcare services in the region [20].

### Design and setting

This cross-sectional study was implemented to better investigate HIV-infected pregnant and postpartum women’s adherence and retention of ART. The study was implemented in eleven public health facilities that provide HIV care, including ART administration. These facilities comprised 3 Municipal Hospitals, 2 District Hospitals, 4 Polyclinics, 1 Maternity Home and 1 Health Centre. These facilities had the highest concentration of HIV-positive residents in the Greater Accra region.

### Target population and sampling procedure

We targeted ART-treated pregnant and postpartum women living with HIV who were at least 18 years old. The actual sample constituted the proportion of the target population who consented to participate and gave written informed consent at the time of their pre- or post-natal visit at any of the eleven healthcare facilities. Convenience sampling was used to select the participants.

In the estimation of the sample size, we used a type I error of 0.025, standardized minimum detectable effect size from a standard normal distribution for adherence (since adherence was our primary outcome) β = 0.26 (equivalent to odds ratio of 1.30) and we simulated 100 to 150 individuals [21, 22]. This sample size of 150 individuals returned a statistical power of 80%. We then adjusted for 30% stopping treatment (as a proxy of loss to follow-up), resulting in a sample size of 176 individuals.

### Data collection instrument

We used a pretested electronic questionnaire for data collection. The questionnaire captured data on socio-demographic characteristics, internalized stigma, anticipated stigma, enacted stigma, social support, ART adherence and uptake and retention in HIV care. The instrument was in the English Language.

### Data collection procedure

Data collection occurred between 29th March and 27th May, 2023 with the aid of seven trained Research Assistants (RAs). The co-principal investigator of the project (IY) was the focal person who supervised the seven RAs. The RAs administered the questionnaire to all participants who consented to participate in the study.

#### Independent variable

Social support was the key independent variable in the study, and this was gauged with a social support score. The parameters used are provided in Table 1. Each question had 4 frequency response options: As much as I would like, less than I would like, much less than I would like, and never, coded as 1,2,3, and 4 respectively. These questions were adapted from the social support index scale [23]. We assessed the reliability of these parameters, and from our sample, the Cronbach alpha was 0.8792. Clearly indicating high internal consistency suggesting that probably the items measure the same underlying concept. The summative score ranged from 9 to 32. These values were categorized into 3 groups using equal width of 8. Hence, values between ‘9–16’indicated high social support, ‘17–24’ represented moderate social support, while values between 25–32 represented low social support.

**Table 1:** Items used for social support score

I get useful advice about important things in my life when I need it.
I get chances to talk to someone about problems at work or with my housework when I need it.
I get chances to talk to someone I trust about my personal and family problems when I need it.
I have people who care what happens to me.
I get love and affection.
I get help with household-related work when I need it.
I get help with money in an emergency when I need it. I get help with transportation when I need it.
I get help when I am sick

In addition, we collected data on socio-demographic characteristics such as participant’s age, marital status, religion, education attainment, place of residence, whether partner’s HIV status is known, and whether participant has a cowife/cowives.

#### Outcome variables

The study had two main outcomes of interest, namely; consistent uptake of ART in the three months preceding the survey and retention in ART. A participant was considered to have been consistent if the person indicated that she took the ART following the prescribed regimen without missing any dose. This category of participants was coded as “1”, whilst those who couldn’t take the dose fully were considered otherwise and coded as “0”. Following the measurement of retention by some existing studies [24–27], retention was measured by asking the participant the last time they visited the hospital to collect their ARV drug. Participants were asked: In the past 3 months, have you taken your ARVs consistently in the correct dosage and at the right time as directed by your medical doctor? The response was “Yes/No”. Those who indicated “Yes” were coded as “1” while those who indicated “No” were coded as “0”.

### Data analysis

Data were analyzed using Stata version 14.1 (College Station, TX). Initial analysis included descriptive statistics characterized by frequencies and percentages to describe the study population. We assessed the associations between social support score, participants’ socio-demographic characteristics, ART adherence within the 3 months preceding the study. This was done using chi-square test, and multivariate associations were assessed using a modified Poisson regression model with health facility clustered robust standard errors. We also examined whether the covariates in the final model modified the relationship between social support and ART adherence. Similarly, associations between social support score, participants’ characteristics, and retention were analyzed using chi-square test and multivariate associations using a modified Poisson regression model with health facility clustered robust standard errors. In model building, we included all variables that had *p*-values of 0.2 or lesser in the bivariate analysis to minimize negative confounding. Overall, a two-sided *p*-value of < 0.05 was considered statistically significant.

### Ethics approval

The Declaration of Helsinki was followed when conducting the study, and two bodies offered ethical approval. Thus, the University of California San Francisco Institutional Review Board and the Ghana Health Service Ethics Review Committee approved the study’s protocol and the informed consent form, with approval numbers 21-35733 and GHS-ERC: 003/12/21 respectively. The authorized permission forms were used by respondents to provide their informed consent before participation. Participants were informed about the study objectives before data collection. We also explained the purpose of the study to all participants, informed them about their rights to participate as well as the right to opt-out from the study at any time without any consequences.

## Results

### Socio-demographic characteristics of the study population

Of the 176 participants who met study eligibility, all of them accepted to participate in the study (response rate 100%). The socio-demographic characteristics of the research participants are presented in Table 2. Median age was 32 years (IQR: 29–36), with the majority of the participants aged 30–34 (36.8%, n = 64) and those below 30 years of age constituting 31.0% (n = 54). More than half of the women were married/separated (55.7%, n = 98) with only 4% (n = 7) being single/never married/had no current partner. Most women knew their partner’s HIV status (63.1%, n = 111) and had co-wives (89.3%, n = 65). A considerable proportion had 1–2 children (47.4%, n = 82), however, 11.0% (n = 19) indicated that they had no children. Three out of ten were identified as Charismatics (32.4, n = 57) and Pentecostals (30.7%, n = 54). Most of the participants had completed primary or less education 60.8% (n = 107), whilst 39.2% (n = 69) had completed O-level or above. ‘About a third (32.4%, n = 57) were working in the informal sector with only 9.1% (n = 16) in the formal sector. Nearly half of the women earned less than 500 GHS (46.0%, n = 81) and 22.2% (n = 39) were not earning any income. When asked to describe their financial status, 77.3% (n = 136) revealed that they were poor. ‘Most of the participants (79.5%, n = 140 and 63.6%, n = 112)’ resided in the urban area and cooked with liquefied petroleum gas (LPG), respectively. About seven out of ten (71.0%, n = 125) had low social support score with only 6.8% (n = 12) recording high social support score.

**Table 2:** Socio-demographic characteristics of the study population

Characteristics	Category	Frequency (%)
Age (*n* = 174)*	Median (IQR)	32 (29–36)
	< 30 years	54 (31.0)
	30–34	64 (36.8)
	> 35 years	56 (32.2)
Marital status	Married/separated	98 (55.7)
	In a relationship, living/not with a partner	71 (40.3)
	Single, never married, no current partner	7 (4.0)
Partner’s HIV status known	No	65 (36.9)
	Yes	111 (63.1)
Have rival/co-wife or co-wives	Yes	18 (10.7)
	No	151 (89.3)
Number of children (*n* = 173)*	None	19 (11.0)
	1–2 children	82 (47.4)
	3 or more children	72 (41.6)
Religion	Protestant/Catholic	33 (18.8)
	Pentecostal	54 (30.7)
	Charismatic	57 (32.4)
	Others (Adventist/Muslim)	32 (18.1)
Education status	Completed primary or less	107 (60.8)
	Completed O level or above	69 (39.2)
Employment status	Formal	16 (9.1)
	Informal	57 (32.4)
	Self-employed	54 (30.7)
	Unemployed	49 (27.8)
Monthly income	Zero income	39 (22.2)
	Below GHc500	81 (46.0)
	GHc500 or more	56 (31.8)
Description of financial status	Poor or not enough	136 (77.3)
	Enough or more than enough	40 (22.7)
Fuel type mainly used	LPG or natural gas	112 (63.6)
	Charcoal/wood	64 (36.4)
Place of residence	Peri-urban/Rural	36 (20.5)
	Urban	140 (79.5)
Social support score	Low	12 (6.8)
	Moderate	39 (22.2)
	High	125(71.0)

*Variables with a lesser sample

### Regression results on social support, socio-demographic characteristics and ART adherence in the 3 months preceding the survey

Bivariate results of social support, socio-demographic characteristics and ART adherence within the 3 months preceding the survey are presented in Table 3. At this level of analysis; age, knowledge of partner’s HIV status, and having rivals/co-wives were significantly associated with ART adherence in the 3 months.

**Table 3:** Bivariate analysis of factors associated with ART adherence in the past 3 months preceding the survey

Characteristics	Category	ART adherence		***p***
		No (%)	Yes (%)	
Social support index	Low	87 (73.7)	38 (65.5)	0.518
	Moderate	24 (20.3)	15 (25.9)	
	High	7 (5.9)	5 (8.6)	
Place of residence	Peri-urban/Rural	29 (24.6)	7 (12.1)	0.053
	Urban	89 (75.4)	51 (87.9)	
Age	< 30 years	28 (23.9)	26 (45.6)	0.013
	30–34	46 (39.3)	18 (31.6)	
	> 35 years	43 (36.8)	13 (22.8)	
Marital status	Married/separated	72 (61.0)	26 (44.8)	0.098
	In a relationship, living/not with a partner	41 (34.8)	30 (51.7)	
	Single, never married, no current partner	5 (4.2)	2 (3.5)	
Partner’s HIV status known	No	36 (30.5)	29 (50.0)	0.012
	Yes	82 (69.5)	29 (50.0)	
Have rival/co-wife or co-wives	No	105 (92.9)	46 (82.1)	0.033
	Yes	8 (7.1)	10 (17.9)	
Number of children	None/refused	10 (8.7)	9 (15.5)	0.323
	1–2 children	54 (47.0)	28 (48.3)	
	3 or more children	51 (44.3)	21 (36.2)	
Religion	Protestant/Catholic	25 (21.2)	8 (13.8)	0.705
	Pentecostal	35 (29.7)	19 (32.8)	
	Charismatic	37 (31.3)	20 (34.5)	
	Others	21 (17.8)	11 (18.9)	
Education status	Completed primary or less	68 (57.6)	39 (67.2)	0.219
	Completed O level or above	50 (42.4)	19 (32.8)	
Employment status	Formal	12 (10.2)	4 (6.9)	0.868
	Informal	37 (31.3)	20 (34.5)	
	Self-employed	35 (29.7)	19 (32.8)	
	Unemployed	34 (28.8)	15 (25.8)	
Monthly income	Zero income	28 (23.7)	11 (19.0)	0.554
	Below GHc500	51 (43.2)	30 (51.7)	
	GHc500 or more	39 (33.1)	17 (29.3)	

In multivariable analysis, two variables had significant relationship with ART adherence, namely age and knowledge of partner’s HIV status (Table 4). Compared to those below 30 years of age, women aged above 35 (aPR = 0.50, CI = 0.35–0.71) had lower prevalence of ART adherence. Similarly, women who knew their partner’s HIV status had lower prevalence of taking ART consistently as compared to those who did not know (aOR = 0.65, CI = 0.45–0.95).

**Table 4:** Multivariable regression model assessing factors associated with ART adherence in the past 3 months preceding the survey

Characteristics	Category	Outcome Present, n (%)	Multivariate analysis	
			PR (95%CI)	***p***
Social support index	Low	5/12 (41.7)	Reference	-
	Moderate	15/39 (38.5)	1.05 (0.51–2.14)	0.898
	High	38/125 (30.4)	0.80 (0.36–1.79)	0.592
Place of residence	Peri-urban/Rural	7/36 (19.4)	Reference	-
	Urban	51/140 (36.4)	1.88 (0.93–3.77)	0.077
Age	< 30 years	26/54 (48.2)	Reference	-
	30–34	18/64 (28.1)	0.60 (0.36–1.02)	0.060
	> 35 years	13/56 (23.2)	0.50 (0.35–0.71)	< 0.001
Marital status	Married/separated	26/98 (26.5)	-	-
	In a relationship, living/not with a partner	30/71 (42.3)	1.12 (0.62–2.01)	0.712
	Single, never married, no current partner	2/7 (28.6)	-	-
Partner’s HIV status known	No	29/65 (44.6)	Reference	-
	Yes	29/111 (26.1)	0.65 (0.45–0.95)	0.026
Have rival/co-wife or co-wives	No	10/18 (55.6)	Reference	-
	Yes	46/151 (30.5)	0.76 (0.54–1.07)	0.122

Although most covariates were insignificant, all the covariates in model in one way modified the relationship between social support and ART adherence. However, the effect of modification was dependent on the level of the covariate considered, for instance being 30 years and below of age modified social support from moderate to high relative to those with low social support. Specifically, those aged 30 years and below had higher prevalence of ART adherence among those with moderate social support compared to those with low, and yet those aged 30–34 years had lower prevalence of ART adherence among those with moderate social support compared to those with low social support. Clearly indicating that age modifies the relationship between social support and ART adherence. We provide more details in the supplementary table S1.

### Results on a modified Poisson regression between social support, socio-demographic characteristics and ART retention

In Table 5, we present the results of the bivariate analysis of social support, socio-demographic characteristic and ART retention. None of the variables in the univariate model showed significant association with retention.

**Table 5:** Bivariate analysis of factors associated with ART retention

Characteristics	Category	Retention		***p***
		No (%)	Yes (%)	
Social support index	Low	68 (68.7)	28 (77.8)	0.533
	Moderate	23 (23.2)	7 (19.4)	
	High	8 (8.1)	1 (2.8)	
Place of residence	Peri-urban/Rural	20 (20.2)	4 (11.1)	0.310
	Urban	79 (79.8)	32 (88.9)	
Age	< 30 years	30 (30.6)	12 (34.3)	0.741
	30–34	38 (38.8)	11 (31.4)	
	> 35 years	30 (30.6)	12 (34.3)	
Marital status	Married/separated	55 (55.6)	19 (52.8)	0.845
	In a relationship, living/not with a partner	40 (40.4)	15 (41.7)	
	Single, never married, no current partner	4 (4.0)	2 (5.6)	
Partner’s HIV status known	No	32 (32.3)	17 (47.2)	0.111
	Yes	67 (67.7)	19 (52.8)	
Have rival/co-wife or co-wives	No	7 (7.4)	6 (17.7)	0.088
	Yes	88 (92.6)	28 (82.3)	
Number of children	None/refused	11 (11.2)	6 (17.1)	0.634
	1–2 children	48 (49.0)	17 (48.6)	
	3 or more children	39 (39.8)	12 (34.3)	
Religion	Protestant/Catholic	20 (20.2)	6 (16.7)	0.535
	Pentecostal	28 (28.3)	15 (41.7)	
	Charismatic	31 (31.3)	9 (25.0)	
	Others	20 (20.2)	6 (16.7)	
Education status	Completed primary or less	63 (63.6)	20 (55.6)	0.394
	Completed O level or above	36 (36.4)	16 (44.4)	
Employment status	Formal	11 (11.1)	3 (8.3)	0.723
	Informal	32 (32.3)	10 (27.8)	
	Self-employed	33 (33.3)	11 (30.6)	
	Unemployed	23 (23.2)	12 (33.3)	
Monthly income	Zero income	21 (21.2)	8 (22.2)	0.879
	Below GHc500	46 (46.5)	18 (50.0)	
	GHc500 or more GHC	32 (32.3)	10 (27.8)	

In the multivariate model, having a rival had significant association with retention such that higher prevalence of ART retention was found among women with rivals relative to those without (aPR = 1.98, CI = 1.16–3.36) as shown in Table 6.

**Table 6:** Multivariable regression model assessing factors associated with retention on ART

Characteristics	Category	Outcome Present, n (%)	Multivariate analysis (*N* = 129)	
			PR (95%CI)	***p***
Social support index	Low	28/96 (29.2)	Reference	-
	Moderate	7/30 (23.3)	0.80 (0.53–1.23)	0.321
	High	1/9 (11.1)	0.32 (0.04–2.95)	0.318
Partner’s HIV status known	No	17/49 (34.7)	Reference	-
	Yes	19/86 (22.1)	0.71 (0.39–1.29)	0.256
Have rival/co-wife or co-wives	No	28/116 (24.1)	Reference	-
	Yes	6/13 (46.2)	1.98 (1.16–3.36)	0.012
Describe your financial status	Poor or not enough	23/103 (22.3)	Reference	-
	Enough or more than enough	13/32 (40.6)	1.77 (0.80–3.90)	0.159

## Discussion

This study investigated the association between social support and ART uptake/retention in Accra, the capital of Ghana. Evidently, social support has no relationship with ART uptake and retention. Our theoretical framework would have expected that women with this support should have higher adherence and retention [17]. This finding suggests that social support, per se, does not guarantee ART uptake and retention among the surveyed pregnant and postnatal women, which possibly implies that other essential factors may rather enhance HIV-infected pregnant and postnatal women’s uptake and adherence to ART. Relatedly, a previous study that investigated illness perceptions, social support and adherence to ART in the Greater Accra region, realized that support from family, friends and other significant others was inversely related to adherence [15].

Similar findings have also been reported from Uganda, as no association was noted between social support (from friends, teachers and classmates) and adherence [11]. Meanwhile, evidence from a systematic review has shown positive impacts of social support toward ART adherence [14]. Additionally, a South African-based study has shown that social support enhances ART adherence and retention [13]. This inconsistency highlights the role of other contextual factors which might present disparate opportunities and bottlenecks to HIV patients in accessing ART, in addition to social support. Our study design did not permit us to unravel the plausible enabling conditions that could foment these women’s motivation for ART, meanwhile, this may be possible when the phenomenon is explored through a qualitative lens. Hence, further research might be required regarding the specific social support that can amplify ART adherence and retention in order to guide workable interventions intending to augment ART adherence and retention, especially as positive association between social support and ART uptake/retention has been realized in other jurisdictions.

The presence of a rival or co-wife was significantly associated with ART retention such that higher odds of ART retention was realised among women with rivals relative to those without rivals. Perhaps these patients are motivated to take good care of themselves to compete with their rivals. Additionally, as advanced by the theoretical framework, women who are co-wives may possess admirable character that attracts genuine social support that consistently motivates them to remain focused on their regimen [17]. The finding put forward that the presence of a rival is associated with increased retention in ART among women living with HIV. Whereas the finding on the association between rivals and retention in HIV care is relatively new, prior research has suggested that social relationships, including both supportive and competitive ones, can have a significant impact on health behaviors and outcomes. For instance, one United States-based study found that social network characteristics, such as size and density, are associated with medication adherence among people living with HIV [28].

Urban residents had higher odds of consistent ART adherence relative to rural/peri-urban residents. This could be as a result of discrepancies in access to healthcare facilities, health education, social support, and other factors [29–32]. Through a sub-Saharan African-based systematic review, Bärnighausen et al. [33] noted that rural residents had lower ART adherence compared to urban residents. Similarly, another study in Kenya indicated that rural residents had lower retention rates in HIV care compared to urban residents [34]. A study conducted in Nigeria also revealed that rural residents tend to have lower levels of HIV knowledge, hence resulting in relatively lower ART adherence [35]. Overall, the finding highlights that interventions aimed at enhancing ART retention and retention should consider the rural/urban dichotomy and the underlying contributory factors, in order to make greater strides.

As compared to women below age 30, those aged 30 and above had lower odds of ART adherence. The finding is indicative that older women living with HIV have lower odds of consistently adhering to ART regimens. Multiplicity of factors might account for this including comorbidities, medication side effects, and socio-economic factors [31, 36]. A systematic review, however, unraveled that ART retention aligns positively with advanced age [37]. It is worth noting that different stages of life present distinct opportunities and limitations to women, hence the critical role of age in ART adherence/retention must always be accorded the requisite recognition in HIV care efforts.

### Strengths and limitations

This is a cross-sectional study and as such, readers are cautioned not to draw causal inferences from this study. Secondly, our principal independent variable was “social support” and there is no absolute measure for this variable. Consequently, the association we found between social support and ART adherence/retention is driven by how we conceptualized it. Additionally, data on “duration on ART” was not collected and yet it is known to influence ART adherence [38]. The study relied on self-reported data other than hospital records, hence there is the possibility that the data was affected by some biases such as social desirability bias. Besides, a convenience sampling approach was used due to nature of the outcome variable and the sample size was relatively low for a quantitative survey. In spite of these limitations, our study uniquely advances the frontiers of knowledge on ART adherence and retention by filling a critical knowledge gap, and offering insightful leads for future HIV interventions.

## Conclusion

Our study showed that social support does not play any essential role in the adherence and retention of ART among the surveyed pregnant and postpartum women. Meanwhile, factors such as urban residency, having a rival and being under age thirty appear to be promising in driving ART adherence and retention. The study has signaled the need for ART adherence/retention scale-up interventions to have a multi-pronged approach in order to identify the multitude of underlying factors that enhance/impede efforts to achieve higher uptake and retention rates. Further investigations may be worthwhile to unravel the specific social support indicators that can yield positive results for ART adherence and retention among pregnant and postnatal women in Accra. Secondly, further engagements with caregivers may be useful in the quest to explore the drivers of HIV antiretroviral therapy in the Accra Metropolis.

### Electronic supplementary material

Below is the link to the electronic supplementary material.

Supplementary Material 1

## Data Availability

materials. The datasets used and/or analyzed during the current study are available from the corresponding author on reasonable request.

## Abbreviations

AIDS: Acquired Immunodeficiency Syndrome
aOR: Adjusted Odds Ratio
ART: Antiretroviral Therapy
CI: Confidence Interval
GHc: Ghana Cedis
GHS: Ghana Health Service
HIV: Human Immunodeficiency Virus
LPG: Liquefied Petroleum Gas
RAs: Research Assistants
WHO: World Health Organisation
MSM: Men who have sex with men
UNAIDS: United Nations Programme on HIV/AIDS
