# Supplementary material for: The role of social support in antiretroviral therapy uptake and retention among pregnant and postpartum women living with HIV in the Greater Accra region of Ghana
Source: BMC Public Health. 2024 Feb 21;24:540. doi: 10.1186/s12889-024-18004-z (PMC10882784; doi:10.1186/s12889-024-18004-z)
Supplement: Supplementary file 1 — Supplementary Material 1 [file 12889_2024_18004_MOESM1_ESM.docx]

**Supplementary Table S1: Adjusted prevalence ratio of the exposure variable in stratified model for each of the covariate stratum**

| **Covariate** | **stratum** | **aPR for social support category** | | |
| --- | --- | --- | --- | --- |
|  |  | low | moderate | High |
| Place of residence | Peri-urban/Rural | reference | 0.88 | - |
|  | Urban | reference | 1.06 | 0.83 |
| Age | <30 years | reference | 1.21 | 0.41 |
|  | 30-34 | reference | 0.89 | 1.64 |
|  | >35 years | reference | 0.89 | 0.84 |
| Marital status | Married/separated | reference | 0.83 | 0.89 |
|  | In a relationship, living/not with a partner | reference | 1.06 | 0.69 |
|  | Single, never married, no current partner | reference | - | - |
| Partner’s HIV status known | No | reference | 1.32 | 0.84 |
|  | Yes | reference | 0.66 | 0.93 |
| Have rival/co-wife or co-wives | No | reference | 1.24 | 0.67 |
|  | Yes | reference | 0.98 | 0.82 |
